# Little associations exist between the three commonly used functional screening tests in collegiate athletes

**DOI:** 10.1038/s41598-024-64518-2

**Published:** 2024-06-13

**Authors:** Mojtaba Asgari, Mohammad Hossein Alizadeh, Mohsen Naderi, Ehsan Abshenas, Mansour Sahebozamani, Shirin Yazdani, Kevin Nolte, Shahab Alizadeh, Mohammadreza Mohammadi, Negar kooroshfard, Ramin Arghadeh, Thomas Jaitner

**Affiliations:** 1 Training and Movement Science Department, Institue for Sport and Sport Science, TU Dortmund University, Otto-Hahn Str.03, 44227 Dortmund, Germany; 2 Department of Health and Sport Medicine, Faculty of Sport Sciences and Health, University of Tehran, Tehran, Iran; 3 Department of Pathology and Corrective Exercise, Faculty of Sport Sciences, Shahid Bahonar University, Kerman, Iran; 4 Department of Motor Control, Faculty of Physical Education and Sport Science, University of Tabriz, Tabriz, Iran; 5 Human Performance Lab, Kinesiology Department, University of Calgary, Calgary, AB Canada; 6 Department of Sport Injuries and Corrective Exercises, Faculty of Sport Sciences, Hakim Sabzevari University, Sabzevar, Iran; 7 Department of Physical Education and Sports Science, Faculty of Educational Sciences and Psychology, Shiraz University, Shiraz, Iran

**Keywords:** Risk factors, Orthopaedics

## Abstract

Although an abundant number of studies have investigated the predictability of the commonly used functional screening tests and despite their popularity and applicability, the relationships between these tests have rarely been studied and have not been well established. This study aimed to examine the potential association between the Functional Movement Screen (FMS), Y Balance Test (YBT), and Landing Error Scoring System (LESS). Six hundred twenty-seven Iranian collegiate athletes (347 males, age = 22.63 ± 4.07, weight = 75.98 ± 13.79, height = 181.99 ± 10.15, BMI = 22.84 ± 3.16; and 280 females, age = 22.22 ± 3.37, weight = 60.63 ± 9.58, height = 166.55 ± 6.49, BMI = 21.81 ± 2.84) participated in this study. Following a 5-min warm-up, each participant underwent a standardized screening battery including the FMS, YBT, and LESS, and the scores were recorded and live coded for the statistical analysis, except for the LESS. The LESS tests were video recorded and scored by one expert examiner using an open-source 2D video analysis software (Kinovea- version 0.9.5), afterwards. The Spearman correlation was utilized as a measure for the correlation, and the Mann‒Whitney U test with a significance level of 0.05 was used to check the differences between male and female athletes. The statistical analysis was performed with RStudio 2023.03.0 using R 4.3.1. A small correlation (0.364) was observed between the FMS composite score and the YBT in male athletes. All other pairwise correlations were negligible among male and female athletes, ranging from − 0.096 to 0.294. Reducing the FMS to the component scores targeting the lower extremities did not alter the correlation to the other screening scores. The median FMS composite score in female athletes was significantly higher than that in males (*p* < 0.001). Negligible correlations exist between the FMS, LESS, and YBT; they do not measure the same values and therefore are irreplaceable with one another. A combination of these tests as a standardized screening battery may potentially better identify injury-predisposed athletes than the application of each test as a stand-alone screening test. Females outperformed males in the FMS test significantly, so sex must be considered a key variable in the FMS studies. Males had slightly higher LESS scores (median difference = 0.5) than females, but this difference is not clinically meaningful. Future research should continue to explore the relationships between various functional screening tests and identify the most effective combinations for comprehensive assessment in different populations and sports disciplines.

## Introduction

Injury prevention and management are fundamental aspects of sport, as injuries can dramatically impair athletes' performance and cause long-lasting physical, psychological, and socioeconomic consequences. A crucial step toward injury prevention is to identify those who are prone to injury. Over the past 15 years, functional screening tests have gained popularity among scientists, practioners, and clinicians as valuable tools for diagnosing movement deficits, identifying potential injury risk factors, and guiding injury prevention strategies. Among these tests, the Functional Movement Screen (FMS), Y Balance Test (YBT), and Landing Error Scoring System (LESS) have been increasingly utilized to evaluate functional movement abilities. They have demonstrate good to exellent interrater (intraclass correlation coefficients (ICC) ranging from 0.84 to 0.93) and intrarater (ICC ranging from 0.87 to 0.91) reliablity^1–3^ and acceptable sensitivity and specificity^4^. They have shown to be practical in discerning deficits in movement behavior and motor function which could contribute to subsequent injuries^1,5–9^. Despite the compromises made for precision that these user-friendly tools entail, they are affordable, easy to operate, and practical to exploit in large-scale settings as alternatives to laboratory measures^3,10–13^.

Each test has specific features and targets different aspects of human movement patterns; the FMS targets seven fundamental movement patterns including balance, mobility and stability, challenging neuromuscular imbalance and movement deficits^6^, the YBT focuses on postural control and lower extremity asymmetry^12^, and the LESS tries to identify risky movement patterns during a jump-landing task^3^. Deficits in the aforementioned functions are considered key risk factors for musculoskeletal injuries^2,3,14–16^. Thus, they serve as user-friendly diagnostic tools, especially in team sports, so injury prevention protocols could be established accordingly^3,10^.

An abundant number of studies have investigated the predictability of these screening tools among active populations^17–21^. Overall, the results are conflicting, making it difficult to derive a clear conclusion regarding the accuracy of these tests to identify at-risk individuals^7,18,19,21,22^.

Despite popularity and applicability, the relationships between these tests have rarely been studied and have not been well established. For instance, Kelleher et al. demonstrated that dynamic postural control measured by the YBT is a very small component of the FMS composite score^23^. De la Motte et al. observed limited overlap in the FMS and YBT and concluded that since both tools assess different risk factors, they should be used together^24^. Conversely, Taylor et al. revealed that the FMS and YBT evaluate similar risk factors, such as dynamic balance and lower extremity power^25^.

The inconsistent findings concerning potential associations between these tests creates uncertainty around whether a combination of screening tests can best identify at-risk athletes and is more beneficial than using a single screening test. Further, understanding the relationship between these tests can provide a more comprehensive picture of an individual's functional movement capacity. While each test is designed to assess a specific aspect of movement, they may complement each other and thus facilitate a more holistic evaluation of the functional performance of an athlete. The findings of Cook et al.^26^ and Pfeifer et al.^27^ emphasizing that the FMS alone is not adequate for injury prediction and that it should be supplemented with other tests and measures of sport readiness support this assumption. Additionally, Šiupšinskas et al. studied a battery of the FMS, YBT, and LESS to predict injury- predisposed female basketball players and suggested that the combination of these tests could be used for more accurate prescreening^28^.

Further, investigating the association between these tests may aid in the development of more targeted injury prevention programs. With a better understanding of the strengths and limitations of each test, users can choose the most appropriate screening tool to identify an individual's specific movement deficits and tailor interventions to tackle them. Such an approach optimizes injury prevention programs and reduces injury risk in sports.

Finally, clarifying the relationship between these tests could also help to identify gaps in current assessment protocols and to develop more effective assessment strategies that benefit athletes and practitioners alike. Therefore, by understanding the associations between the FMS, YBT, and LESS, we have an opportunity to better evaluate functional movement patterns, optimize injury prevention and intervention strategies, and ultimately enhance athlete health and performance. Given the existing lack of experimental evidence regarding the association between these tests and the need for a comprehensive assessment of an individual's functional movement capacity, the current study aims to address the associations between three commonly used tests, namely, the FMS, the YBT, and the LESS. Additionally, this investigation aims to provide insight into the interoperability and potential joint use of these tests. Ultimately, since sex has been labeled a key risk factor influencing functional assessments^29^, we also address any potential associations across sexes as we expect that males perform differently from females in all tests. We assume that the FMS, YBT, and LESS do not measure the same values and are not associated.

## Methods

### Study design and participants

Through a cross-sectional study design, we examined the potential relationships between the FMS, YBT, and LESS scores. At baseline, the participants' characteristics, such as age, height, weight, BMI, foot length, footedness, sport, and level of competition, were recorded to ensure a diverse representation of athletes from various disciplines. Six hundred thirty-two Iranian collegiate athletes (352 males, age = 22.63 ± 4.07, weight = 75.98 ± 13.79, height = 181.99 ± 10.15, BMI = 22.84 ± 3.16; and 280 females, age = 22.22 ± 3.37, weight = 60.63 ± 9.58, height = 166.55 ± 6.49, BMI = 21.81 ± 2.84) participating in the 15th Iranian National University Olympiad agreed to participate in this study.

### Ethics, consent, and inclusion criteria

The present study was conducted in accordance with the Helsinki declaration guidelines. Participation was voluntary, and ethical approval was obtained from the Research Ethics Committee of the Sport Sciences Research Institute of Iran (IR.SSRI.REC.1400.1334). Prior to data collection, the study's purpose, procedures, and potential risks were explained to the participants, and written informed consent was obtained from all participants.

Participants were eligible for inclusion if they met the following criteria: (1) collegiate athletes actively participating in their sports, (2) registered as a university athlete participating in the Olympiad, and (3) no current musculoskeletal injuries or health problems such as respiratory infection, recent concussion history, or Otolith organs’ problems that would impede participation in the screening tests (by answering “no” to all of the questions in the Physical Activity Readiness Questionnaire)^30^.

### Procedures

Following a 5-min warm-up, each participant underwent the FMS, YBT, and LESS in one testing session. The order of administration for the tests was randomized to minimize any potential order effects. They were not familiarized with the test battery prior to testing, as knowledge of the scoring schemes seems to affect performance^31^. The testing sessions were conducted by trained researchers who were experienced in administering and scoring the functional screening tests.

### FMS

The FMS was conducted according to the standardized protocol outlined by Cook et al.^6^. Participants performed a series of seven fundamental movement patterns, including deep squat, hurdle step, inline lunge, shoulder mobility, active straight leg raise, trunk stability push-up, and rotary stability. Each movement pattern was scored on a scale from zero to three based on the presence of movement dysfunctions or limitations. Each athlete performed a maximum of three trials for each FMS task. If one met the criteria for a score ‘three’ before completing all trials for a particular task, they proceeded to the next task, as additional trials was unnecessary. For the FMS, the best scores were live recorded and considered for further analysis. The total FMS score ranged from zero to 21, with higher scores indicating better movement quality. Additionally, deep squat, hurdle step, inline lunge and active straight leg raise were considered the FMS components targeting the lower extremities and were analyzed separately with a score between zero and twelve.

### YBT

The YBT was conducted following the procedures established by Plisky et al.^12^. While standing on one leg with the other leg reaching as far as possible along a measuring tape, each participant performed six practice trials as suggested in anterior, posteromedial, and posterolateral directions (with shoes off) to account for the learning effect and the greatest of three test trials was used for analysis^12,32^. The distance reached was measured by a measuring tape and normalized to the athlete’s leg length. Then, the average of the three measures was calculated for each leg and subsequently summed as the YBT performance measure.

### LESS

The LESS test was performed identical to the method proposed by Padua et al.^3^. A 30-cm height box was adjusted at a distance of 50% of each participant’s height to the landing line. Two digital video camera recorders (Sony HD 24.5 mega pixel, HDR- PJ810E) were set up in the frontal and sagittal views to the landing direction to capture the movement. The players were instructed to stand on the box while wearing sport shoes and jump off the box with both feet over the landing line and rebound for a maximal vertical jump immediately after landing. If anyone failed to jump with both feet simultaneously, jumped off the box visibly in the vertical direction, did not pass the landing line, or ultimately could not complete the task smoothly, the respective trial was repeated. Each participant performed three trials and the average score was considered for the statistical analysis.

The LESS tests were video recorded and scored afterwards by a single expert examiner with a between-day reliability of 0.95% ICC to blind the potential of interrater reliability bias. The open-source 2D software Kinovea (version 0.9.5; http://www.kinovea.org) was used to determine the ankle, knee, hip, and trunk angles from both sagittal and frontal planes.

### Data analysis and statistical analysis

For the statistical analysis, the ordinal scaling of the FMS and LESS needs to be considered. Therefore, the descriptive analysis shows median and interquartile range (iqr) for central tendency and variability. The Mann‒Whitney U test with a significance level of 0.05 was used to check if differences between male and female athletes were apparent within the aforementioned screening tests. This lead to analyzing male and female athletes separately in the subsequent analysis. For correlation analysis, the Spearman correlation was calculated, and the outcomes were interpreted according to the descriptions for absolute values: negligible (0–0.3), small (0.3–0.5) moderate (0.5–0.7), high (0.7–0.9), and very high (0.9–1)^33^. The bootstrap method was used to create 1000 samples from the original data^34^. Performing the correlation analysis on all of these samples allows the estimation of empirical confidence intervals for the Spearman correlation without assuming a theoretical distribution by calculating the respective percentiles of the 1000 samples.

Further, we quantify the absolute number of athletes classified as injury-prone, utilizing established cutoff scores for the FMS and the LESS in practical applications settings^19,20^. We compare the percentages of injury-prone athletes identified solely by each test and those identified by both tests in combination. This comparison, alongside correlation analysis, enable us to assess the unique identification of at-risk athletes by each test, their intersection, and provide additional insights into the concordance between the tests, thus enhancing our understanding of their validity. The statistical analysis was performed with RStudio 2023.03.0 using R 4.3.1.

## Results

Male and female athletes showed similar descriptive results in central tendency and variability for the YBT and LESS, and no significant differences were found (Table 1). Overall, the median FMS total score in female athletes was two points higher than that in males, and a significant difference was observed between sexes (Table 1).

**Table 1:** Median and interquartile range (iqr) of the screening too scores for male and female athletes and results of the Mann‒Whitney U test for differences.

Screening test	Females	Males	*p* Value
FMS	17 (2)	15 (3)	< 0.001*
YBT	176.7 (22.4)	173.1 (20.6)	0.928
LESS	3.5 (4)	4 (4)	0.703

*Significant difference.

A small correlation (0.364) was observed between the FMS total score and the YBT in male athletes. All other pairwise correlations were negligible for both male and female athletes, ranging from − 0.096 to 0.294^33^. Reducing the FMS to the component scores targeting the lower extremities did not alter the correlation to the other screening scores. The largest change was a reduction of 0.07 in the correlation to the YBT for male athletes (Table 2).

**Table 2:** Spearman correlations and empirical confidence intervals (CI) between screening scores. FMS lower extremities is the sum of scores in deep squat, hurdle step, in-line lunge and active single leg raise.

Screening test	Female		Male	
	LESS	YBT	LESS	YBT
FMS	− 0.045 [− 0.239; 0.129]	0.253 [0.135; 0.362]	0.147 [0.019; 0.258]	0.364 [0.265; 0.459]
FMS lower extremities	− 0.031 [− 0.217; 0.156]	0.241 [0.126; 0.345]	0.099 [− 0.045; 0.224]	0.294 [0.189; 0.392]
YBT	− 0.096 [− 0.268; 0.090]		0.186 [0.052; 0.305]	

In our analysis across sports, we found negligible to moderate associations between the tests ranging from − 0.186 [CI − 0.687; 0.359] to 0.405 [CI 0.080; 0.662] in 14 different sports including basketball, volleyball, futsal, handball, table tennis, swimming, wrestling and track and field. Meanwhile, we observed high correlations between the FMS and YBT among badminton 0.748 [CI 0.432; 0.912] and combat sports 0.748 [CI 0.432; 0.912] athletes.

## Discussion

This is the first large-scale study concerning the association between three commonly used functional screening tests and provides practical evidence behind the potential utility of applying the FMS, YBT, and LESS tests as an integrated screening battery. The lack of association between these tests demonstrates that they potentially capture different dimensions of movement quality, may not be interchangeable in assessing functional performance, are irreplaceable with one another, and might likely better identify at-risk athletes when utilized as a standardized screening battery. Athletes who perform well in one test may not necessarily excel in the others, suggesting the need for a comprehensive screening approach that combines multiple tests and tackles a broader spectrum of functional abilities. In other words, the LESS might identify at-risk individuals overlooked by the FMS and the YBT, and vice versa. This underscores the diverse movement dimensions evaluated by these tests, each recognizing distinct deficits as potential injury risks. These findings are in line with Cook et al., Pfeifer et al., and Kelleher et al.^23,26,27,35^, supporting the efficacy of using a combination of functional tests for identifying at-risk athletes. In contrast, our study contradicts the findings of Taylor et al., showing that the FMS and YBT assess similar underlying measures in high school students^25^. Our findings have implications for practitioners applying functional screening tests in athletic settings, suggesting that employing a battery of screening tests, including the FMS, YBT, and LESS, might provide a more holistic assessment of movement abilities.

Consistent with this study, Lisman et al. found no significant associations between the presence of lower extremity asymmetry measured by the YBT and a low score (score of 1) on any component of the FMS test^36^. Brumitt et al. realized that a battery of screening tests involving standing long jump (SLJ), single-leg hop (SLH), and the lower extremity functional test (LEFT) could be useful for identifying at-risk female athletes^37^. Further, Taylor et al. reported that there is a need for the application of multiple field-based tests to identify athletes’ movement and physical performance characteristics^25^. Hence, incorporating screening tests into an integrated screening battery might improve the potential identification of injury-predisposed individuals by challenging the majority of risk factors. In line with this assumption, Šiupšinskas et al. found that the combination of the FMS, YBT, and LESS tests appears to be more beneficial for preparticipation assessment than the application of a single screening test^28^.

The specific nature and structure of each test indicates why they are not correlated and cannot measure the same values. Both the YBT and LESS tests are proposed to identify certain movement deficits e.g., lower extremity asymmetry and malalignments during a jump-landing task, respectively^38–40^, while the FMS is a full-body screening tool that reflects fundamental proprioception and kinesthetic awareness principles^6,8,9^. Based on such a simple explanation, we may conclude that although the YBT is a reliable and valid test for assessing postural control and asymmetry, but it is ineligible for the assessment of risky movement patterns in highly dynamic conditions that may contribute to an increased likelihood of sustaining injury, although postural control is a component of a proper landing. In other words, an individual might have excellent postural control but still be at high risk of injury due to improper landing biomechanics, muscle imbalances, dysfunctions, and lack of stability or mobility. Findings of Walbright et al. wherein they observed lack of validity to predict lower quarter injury risk in female collegiate athletes support this assumption^41^. Thus, integrating FMS, YBT, and LESS tests would potentially increase the applicability of the screening process and minimize the probability of missing at-risk individuals. Athletes may exhibit unique movement patterns and compensatory strategies that are not consistently captured by all three tests, leading to the absence of significant associations.

Further, the outcomes of this study show that the median FMS composite score in female athletes is significantly higher than that in males, which is in line with the studies of Paszkewicz et al.^42^ and Knapik et al.^43^ and partially supports the outcomes of Taylor et al. reporting that females outperform males on the FMS and YBT^25^. Our findings, on the other hand, contradict the outcomes of Abraham et al., suggesting that U17 male athletes slightly outperform U17 female athletes on the FMS test^44^. Moore et al. reviewed the factors influencing the correlation between the FMS and injury risk and illustrated that sex is a fundamental variable in the FMS studies^45^. Additionally, Gnacinski et al. addressed a sex bias in the FMS literature and found that the FMS aggregate score is not equally meaningful for male and female populations^46^. Therefore, there is strong evidence confirming that sex significantly affects the FMS scores, and adapting cutoff values based on sex would be beneficial in increasing the quality of screening by increasing sensitivity. In this regard, Bahr, by scrutinizing the functional screening literature, emphasized that for a successful screening plan, it is essential to consider sex and history of injury^29^. Additionally, Lehr et al. assessed the risk of non-contact injury based on the YBT and recommended that injury risk should be determined based on sport, sex, and age^17^.

The most recent meta-analysis across the LESS literature reveals that males have significantly lower LESS scores than females and suggests that sex might impress the LESS, although the difference of 0.6 errors is not clinically meaningful^20^. The current outcomes, on the other hand, demonstrate that females have slightly lower LESS scores (median difference = 0.5) than males, but this difference is not clinically meaningful either and reaffirms the previous studies of Smith et al.^47^, Lam et al.^48^, Welling et al.^49^, DiStefano et al.^50^, and Jacobs et al.^51^. Given the available inconsistency and that the quality of previous studies on the LESS was identified to be low, further well-established studies are required to address whether sex truly influences the LESS scores. In this regard, two previous studies illustrated that mid-to-long term application of the 11+ warm up program develops landing patterns and reduces the LESS scores in male football players^52^ but does not improve the landing pattern and the LESS scores in preadolescence female players^53^.

### Perspective

Our findings offer primary scientific evidence supporting the utility of a standardized screening battery including the FMS, YBT, and LESS, and contributes to the field by demonstrating that the commonly used screening tests capture different movement patterns, are irreplaceable with one another, and may potentially better identify athletes who are prone to injury when utilized as a standardized screening battery. The results emphasize the importance of implementing these tests as part of routine screening protocols by practitioners, sports medicine professionals, and trainers.

Further, our study sheds light on the presence of sex differences within the FMS literature, indicating that sex should be considered a key factor influencing field-based screening outcomes. This illustrates the need for tailored assessment approaches that account for sex-specific characteristics and movement patterns. By recognizing these differences, practitioners can refine their screening strategies and interventions, ultimately optimizing injury prevention and performance enhancement programs for both male and female athletes.

## Conclusion

The associations between the FMS, YBT, and LESS tests in both male and female collegiate athletes are mostly negligible. The combination of these tests may potentially create a comprehensive user-friendly screening battery that maximizes the applicability of functional screenings to identify those who are prone to injury. Further prospective studies to address the ability of that screening battery to predict at-risk individuals are needed. Additionally, future research should continue to explore the relationships between various functional screening tests and identify the most effective combinations for comprehensive assessment in different populations and sports disciplines. The mean FMS composite score in female athletes is significantly higher than that in males, indicating that sex is a key factor influencing the FMS scores. Females have slightly lower LESS scores (0.5) than males, but this difference is not clinically meaningful.

## Limitations

Limitations of this study include the cross-sectional design, which does not establish causality or the long-term predictive value of these tests. Moreover, the specific characteristics of the study population, such as being collegiate athletes, may limit the generalizability of the findings to other populations.

## Data Availability

The datasets generated and analysed during this study are available from the corresponding author upon request.
